# U-Shaped Relationship of Non-HDL Cholesterol With All-Cause and Cardiovascular Mortality in Men Without Statin Therapy

**DOI:** 10.3389/fcvm.2022.903481

**Published:** 2022-07-07

**Authors:** Rui-Xiang Zeng, Jun-Peng Xu, Yong-Jie Kong, Jia-Wei Tan, Li-Heng Guo, Min-Zhou Zhang

**Affiliations:** ^1^The Second Clinical College of Guangzhou University of Chinese Medicine, Guangzhou, China; ^2^Department of Critical Care Medicine, Guangdong Provincial Hospital of Chinese Medicine, Guangzhou, China

**Keywords:** non-HDL cholesterol, all-cause mortality, cardiovascular mortality, U-shaped relationship, men

## Abstract

**Background:**

Non-HDL-C is well established causal risk factor for the progression of atherosclerotic cardiovascular disease. However, there remains a controversial pattern of how non-HDL-C relates to all-cause and cardiovascular mortality, and the concentration of non-HDL-C where the risk of mortality is lowest is not defined.

**Methods:**

A population-based cohort study using data from the National Health and Nutrition Examination Survey (NHANES) from 1999 to 2014. Male participants without statin therapy were divided into the six groups according to non-HDL-C levels (<100, 100–129, 130–159, 160–189, 190–219, ≥220 mg/dl). Multivariable Cox proportional hazards models were conducted with a hazard ratio (HR) and corresponding 95% confidence interval (CI). To further explore the relationship between non-HDL-C and mortality, Kaplan–Meier survival curves, restricted cubic spline curves, and subgroup analysis were performed.

**Results:**

Among 12,574 individuals (average age 44.29 ± 16.37 years), 1,174(9.34%) deaths during a median follow-up 98.38 months. Both low and high non-HDL-C levels were significantly associated with increased risk of all-cause and cardiovascular mortality, indicating a U-shaped association. Threshold values were detected at 144 mg/dl for all-cause mortality and 142 mg/dl for cardiovascular mortality. Below the threshold, per 30 mg/dl increase in non-HDL-C reduced a 28 and 40% increased risk of all-cause (*p* < 0.0001) and cardiovascular mortality (*p* = 0.0037), respectively. Inversely, above the threshold, per 30 mg/dl increase in non-HDL-C accelerated risk of both all-cause mortality (HR 1.11, 95% CI 1.03–1.20, *p* = 0.0057) and cardiovascular mortality (HR 1.30, 95% CI 1.09–1.54, *p* = 0.0028).

**Conclusions:**

Non-HDL-C was U-shaped related to all-cause and cardiovascular mortality among men without statin therapy.

## Introduction

Non-high-density lipoprotein cholesterol (non-HDL-C), which represents the total cholesterol content of apolipoprotein B-containing lipoproteins, includes very low-density lipoproteins (VLDL) and their metabolic remnants, intermediate-density lipoproteins (IDL), lipoprotein(a), and low-density lipoproteins (LDL) (1, 2). Non-HDL-C is well established causal risk factor for the development of atherosclerotic cardiovascular disease (1, 3, 4). Two decades ago, non-HDL-C was highlighted as an important secondary lipid therapeutic goal in the United States National Cholesterol Education Program's Adult Treatment Panel (5). Furthermore, the National Lipid Association and International Atherosclerosis Society recently recommended that non-HDL-C should be an equal target to LDL-cholesterol (LDL-C) in patients with atherosclerotic cardiovascular disease (6). Preponderantly, non-HDL-C trajectories remain fairly flat, and non-HDL-C between age 25 and 40 years is sufficient to confidently categorise individuals as high or low non-HDL-C for the next 25 to 30 years (3). Moreover, non-HDL-C can be accurately calculated in a non-fasting specimen, without incurring additional expense (7).

A systematic review and meta-analysis by our team previously demonstrated that the increased levels of non-HDL-C were significantly associated with an increased risk of mortality in patients with coronary heart disease (CHD) (8), which was similar to previous research findings in population without cardiovascular diseases (9) or patients with diabetes (10). Interestingly, a U-shaped relationship was also recently identified in different populations (11, 12). Studies detected the U-shaped association of non-HDL-C with all-cause and cardiovascular mortality among patients with hypertension or chronic kidney disease (CKD) stages 3–5 (11, 12), non-HDL-C was U-shaped associated with mortality among male hypertension in subgroup analysis, but not in female (12). Differently, one study focused on the men population, from the Israeli National Death Registry demonstrated a positive association that non-HDL-C as a useful predictor of cardiovascular disease mortality in 22 years follow-up (13). Hence, for more clearly understand how non-HDL-C relates to all-cause and cardiovascular mortality in men, the aim of the present study used data from the large population representative surveys to determine the relationship of non-HDL-C with all-cause and cardiovascular mortality, and the concentration of non-HDL-C associated with the lowest risk of mortality in men without statin therapy.

## Materials and Methods

### Study Population

We performed a population-based cohort study using data from the National Health and Nutrition Examination Survey (NHANES), and data were combined across 8 continuous NHANES cycles: 1999–2000, 2001–2002, 2003–2004, 2005–2006, 2007–2008, 2009–2010, 2011–2012, and 2013–2014. NHANES is a series of national surveys to evaluate the health status of the United States population with a complex, stratified, multistage, probability sampling method. The Centres for Disease Control and Prevention ratified the study protocols, and all the participants provided written informed consent. Detailed about the NHANES has been published elsewhere (14, 15).

**Figure 1:**
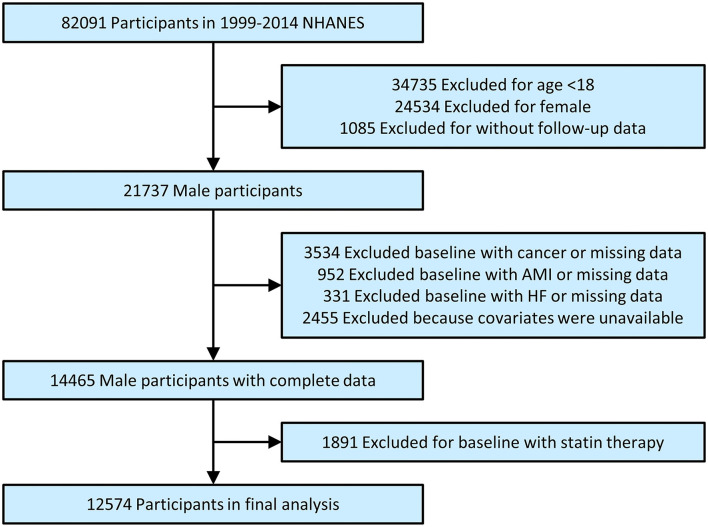
Study flowchart. NHANES, National health and nutrition examination survey; AMI, acute myocardial infarction; HF, heart failure.

The total number of participants in primary survey was 82,091. After excluding participants for age <18 (*n* = 34,735), female (*n* = 24,534) or without follow-up data (*n* = 1,085), and those in baseline with cancer or missing data (*n* = 3,534), acute myocardial infarction (AMI) or missing data (*n* = 952), heart failure (HF) or miss data (*n* = 331) and excluding because covariates were unavailable (n = 2,455), finally excluding for baseline with statin therapy (*n* = 1,891). Hence, 12,574 individuals were included in our final analysis (Figure 1).

### Covariates

Fasting samples obtained from peripheral venous blood were stored at −20°C and shipped weekly for laboratory analyses. Non-HDL-C levels were calculated from total cholesterol (TC) minus HDL cholesterol (HDL-C). The measurement of TC used with an enzymatic assay method, and HDL-C was used with a heparin-manganese precipitation method or a direct immunoassay technique. Further detailed information about the collection of blood samples and lipid concentration measurement is available in another study (16). In addition, creatinine, haemoglobin, and glycated haemoglobin A1c (HbA1c) measurements were based on standardised procedures. Demographic variables such as age, gender, body weight, height, race/ethnicity (Mexican American, other Hispanic, non-Hispanic White, non-Hispanic Black, other race), education (Lower than high school, high school, more than high school), were acquired according to the household interview. Information on smoking status (current smoker, former smoker, and never smoked), and history of disease had been assessed at baseline by standard examinations, and questionnaires were administered by trained health technicians, interviewers, and physicians. The mean blood pressure was calculated as the average of three valid measurements. Nutritional status (e.g., energy intake, protein intake, carbohydrate intake, total fat intake) was acquired according to the dietary interview. A “multiple pass” 24-h dietary interview format was used to collect detailed information about all foods and beverages, which were used to estimate the total intake of energy, nutrients, and non-nutrient food components. More information is available at www.cdc.gov/nchs/nhanes.

### Outcomes

The primary determination of mortality for eligible participants is based upon matching survey records to the records from the National Death Index (NDI), and other sources including the Social Security Administration, the Centres for Medicare and Medicaid Services, data collection, and for the follow-up surveys of the National Centre for Health Statistics, ascertainment of death certificates are also incorporated. Participants were eligible for mortality follow-up based on matching identifying information during their NHANES interviews, such as the last 4 digits of social security number, full name, date of birth, state of birth, state of residence, marital status, race, and sex (17). All-cause mortality and cardiovascular mortality were the endpoints of the present study. The mortality status of individuals was obtained from data from the NDI through December 31, 2015. This study classified causes of mortality referred to the codes of the international statistical classification of diseases, 10th revision (ICD-10). Cardiovascular mortality was defined by the ICD-10 codes for as I00-I09, I11, I13, and I20-I51. When treating cardiovascular mortality as an outcome, the deaths due to other causes were censored.

### Statistical Analysis

The data were presented as mean values with standard deviation (SD), the median with interquartile ranges, or frequencies with percentages, as appropriate. Comparisons of the differences between groups were made with one-way ANOVA, chi-square tests, or Kruskal–Wallis H-tests by the classification of non-HDL-C levels (<100, 100–129, 130–159, 160–189, 190–219, ≥220 mg/dl). Survival analysis according to non-HDL-C stratification was performed using standardised Kaplan–Meier curves. The proportional hazard assumption was examined and met by plotting the log minus log survival curves and survival times. The multivariable Cox proportional hazards models were used for exploring the association of non-HDL-C with all-cause and cardiovascular mortality. In model 1, there was no adjustment. In model 2, we adjusted for age and race. In model 3, we adjusted for age, race, education, body mass index (BMI), systolic blood pressure, diastolic blood pressure, smoking, diabetes, hypertension, CHD, stroke, creatinine, haemoglobin, HbA1c, triglycerides, energy intake, protein intake, carbohydrate intake, and total fat intake. Restricted cubic spline models were used for nonlinear relationships with knots at 5, 35, 65, and 95 percentiles of non-HDL-C. If the relationships were non-linear, the difference of relationships at the threshold was detected by two piecewise linear regression models. The point with the highest likelihood among all the possible values was chosen to define the threshold value. The differences in the results when applying one-line or two piecewise linear regression models were compared by a logarithmic likelihood ratio test. Furthermore, the subgroup analysis includes age (<65 or ≥65 years), race (White, Black, or other race), education (lower than high school, high school, more than high school), BMI (<25 or ≥25 kg/m2), smoking (yes or no), diabetes (yes or no), hypertension (yes or no). All statistical analyses were performed using R version 3.3.2 (R Foundation for Statistical Computing, Vienna, Austria), and *p* < 0.05 was considered statistically significant.

## Results

### Baseline Characteristics

Finally, 12,574 individuals (average age 44.29 ± 16.37 years) were included in this analysis. Table 1 demonstrates baseline characteristics according to non-HDL-C levels. There were significant subgroup differences in age, race, education, BMI, systolic blood pressure, diastolic blood pressure, smoking, hypertension, haemoglobin, HbA1C, triglycerides, TC, HDL-C, LDL-C, energy intake, and carbohydrate intake (all *p* < 0.001), except for diabetes, CHD, stroke, creatinine, protein intake, total fat intake, all-cause, and cardiovascular mortality. We found 1,174 (9.34%) all-cause deaths and 184 (1.46%) cardiovascular deaths during a median follow-up of 98.38 ± 53.78 months.

**Table 1:** Baseline characteristics according to non-HDL-C levels.

		**Non-HDL-C, mg/dl**						***P*** **-value**
	**Total**	**<100**	**100–129**	**130–159**	**160–189**	**190–219**	**≥220**	
N	12,574	1,298	2,690	3,644	2,786	1,400	756	
Age, years	44.29 ± 16.37	36.87 ± 16.98	41.77 ± 17.42	45.24 ± 16.47	46.79 ± 15.43	46.93 ± 14.10	47.26 ± 12.92	<0.001
Race								<0.001
White	5,562 (44.23%)	534 (41.14%)	1,138 (42.30%)	1,671 (45.86%)	1,230 (44.15%)	626 (44.71%)	363 (48.02%)	
Black	2,539 (20.19%)	437 (33.67%)	663 (24.65%)	685 (18.80%)	461 (16.55%)	185 (13.21%)	108 (14.29%)	
Other	4,473 (35.57%)	327 (25.19%)	889 (33.05%)	1,288 (35.35%)	1,095 (39.30%)	589 (42.07%)	285 (37.70%)	
Education								<0.001
Lower than high school	3,565 (28.35%)	322 (24.81%)	738 (27.43%)	977 (26.81%)	863 (30.98%)	417 (29.79%)	248 (32.80%)	
High school	3,027 (24.07%)	330 (25.42%)	645 (23.98%)	863 (23.68%)	644 (23.12%)	361 (25.79%)	184 (24.34%)	
More than high school	5,982 (47.57%)	646 (49.77%)	1,307 (48.59%)	1,804 (49.51%)	1,279 (45.91%)	622 (44.43%)	324 (42.86%)	
Body mass index, kg/m^2^	28.09 ± 5.69	25.30 ± 5.21	26.92 ± 5.94	28.47 ± 5.84	29.04 ± 5.37	29.29 ± 4.96	29.44 ± 4.91	<0.001
SBP, mmHg	124.19 ± 16.20	120.82 ± 15.59	122.57 ± 15.83	124.33 ± 16.35	125.22 ± 16.35	126.02 ± 15.49	127.92 ± 16.89	<0.001
DBP, mmHg	72.83 ± 11.86	68.65 ± 11.77	70.76 ± 11.60	72.77 ± 11.89	74.34 ± 11.25	75.41 ± 11.65	77.21 ± 11.86	<0.001
Smoking	6,673 (53.07%)	660 (50.85%)	1,378 (51.23%)	1,888 (51.81%)	1,456 (52.26%)	810 (57.86%)	481 (63.62%)	<0.001
Diabetes	765 (6.08%)	68 (5.24%)	171 (6.36%)	208 (5.71%)	171 (6.14%)	85 (6.07%)	62 (8.20%)	0.116
Hypertension	2,921 (23.23%)	217 (16.72%)	568 (21.12%)	867 (23.79%)	695 (24.95%)	359 (25.64%)	215 (28.44%)	<0.001
Coronary heart disease	99 (0.79%)	8 (0.62%)	26 (0.97%)	22 (0.60%)	27 (0.97%)	9 (0.64%)	7 (0.93%)	0.428
Stroke	178 (1.42%)	9 (0.69%)	33 (1.23%)	57 (1.56%)	41 (1.47%)	23 (1.64%)	15 (1.98%)	0.133
Creatinine, mg/dl	86.46 ± 34.07	87.25 ± 33.31	86.99 ± 31.75	85.80 ± 26.28	86.57 ± 30.04	86.60 ± 55.42	85.77 ± 38.44	0.689
Haemoglobin, g/l	15.23 ± 1.18	14.82 ± 1.30	15.07 ± 1.24	15.24 ± 1.16	15.35 ± 1.11	15.46 ± 1.05	15.54 ± 1.14	<0.001
HbA1c, %	5.58 ± 0.99	5.33 ± 0.72	5.46 ± 0.84	5.55 ± 0.89	5.63 ± 0.94	5.75 ± 1.18	6.08 ± 1.67	<0.001
Triglyceride, mg/dl	124.04 (82.04–198.02)	66.01 (49.00–92.06)	92.06 (66.98–131.04)	122.00 (86.03–176.05)	156.02 (108.00–231.07)	196.07 (134.05–293.09)	265.58 (178.09–456.62)	<0.001
TC, mg/dl	199.05 ± 42.07	139.13 ± 20.04	167.38 ± 16.61	192.14 ± 15.25	218.15 ± 14.29	245.97 ± 13.75	290.67 ± 42.03	<0.001
HDL-C, mg/dl	47.84 ± 14.00	55.01 ± 17.04	51.57 ± 15.05	47.56 ± 13.19	44.94 ± 11.96	43.74 ± 11.35	41.96 ± 10.90	<0.001
LDL-C, mg/dl	121.54 ± 35.37	69.56 ± 12.96	95.66 ± 12.49	118.73 ± 14.48	142.30 ± 14.99	165.13 ± 16.73	202.66 ± 38.87	<0.001
Non-HDL-C, mg/dl	151.21 ± 43.54	84.13 ± 12.14	115.81 ± 8.54	144.57 ± 8.65	173.22 ± 8.48	202.23 ± 8.50	248.70 ± 41.75	<0.001
Energy intake, kcal	2,562.21 ± 1,148.91	2,698.28 ± 1,208.16	2,599.52 ± 1,201.38	2,554.34 ± 1,129.65	2,497.82 ± 1,080.05	2,537.78 ± 1,172.34	2,516.30 ± 1,127.45	<0.001
Protein intake, gm	98.00 ± 49.24	100.23 ± 53.75	99.25 ± 51.72	97.09 ± 47.13	96.43 ± 47.57	99.31 ± 48.32	97.44 ± 49.75	0.088
Carbohydrate intake, gm	307.40 ± 147.76	326.10 ± 155.89	310.65 ± 152.37	308.76 ± 148.86	299.71 ± 137.13	301.92 ± 151.54	295.73 ± 138.69	<0.001
Total fat intake, gm	94.34 ± 53.27	97.27 ± 55.11	95.99 ± 55.48	93.91 ± 52.47	92.72 ± 50.79	93.45 ± 53.11	93.17 ± 54.83	0.073
All-cause mortality	1,174 (9.34%)	115 (8.86%)	258 (9.59%)	312 (8.56%)	277 (9.94%)	134 (9.57%)	78 (10.32%)	0.381
Cardiovascular mortality	184 (1.46%)	21 (1.62%)	39 (1.45%)	41 (1.13%)	41 (1.47%)	23 (1.64%)	19 (2.51%)	0.101

*Non-HDL-C, non-high-density lipoprotein cholesterol; N, number; SBP, systolic blood pressure; DBP, diastolic blood pressure; HbA1c, glycated haemoglobin A1c; TC, total cholesterol; HDL-C, high-density lipoprotein cholesterol; LDL-C, low-density lipoprotein cholesterol*.

*Values are expressed as the mean ± SD, the median with interquartile range or n (%)*.

### Relationships of Non-HDL-C With All-Cause and Cardiovascular Mortality

**Figure 2:**
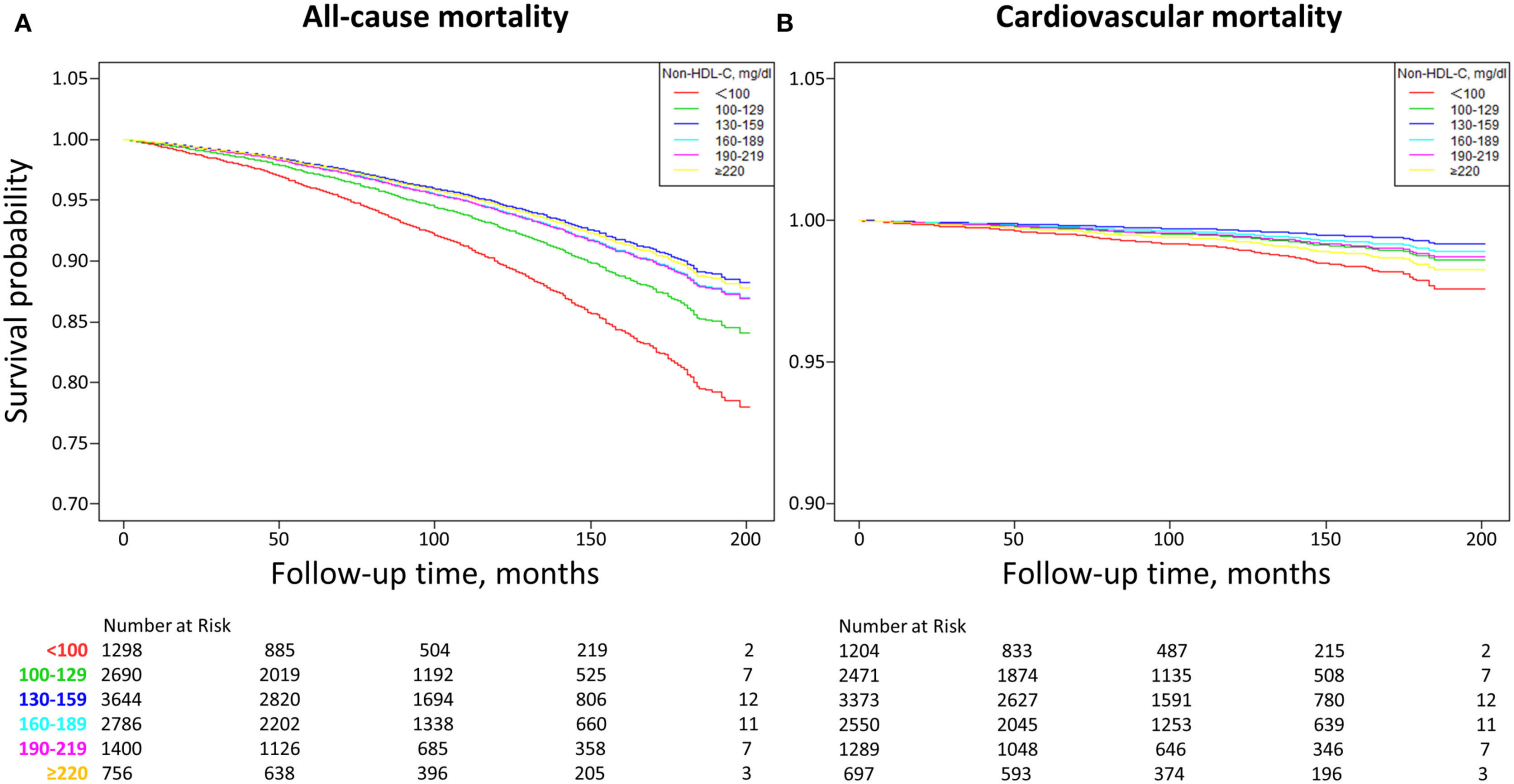
Kaplan-Meier survival curves of non-HDL-C with all-cause **(A)** and cardiovascular **(B)** mortality. Non-HDL-C, non-high-density lipoprotein cholesterol. **(A)** Other groups vs. non-HDL-C <100 mg/dl (all *P* < 0.005). **(B)** non-HDL-C = 130–159 or 160–189 mg/dl vs. non-HDL-C <100 mg/dl (all *P* < 0.005), non-HDL-C = 100–129 mg/dl vs. non-HDL-C <100 mg/dl (*P* = 0.0443), non-HDL-C = 190–219 mg/dl vs. non-HDL-C <100 mg/dl (*P* = 0.0546), non-HDL-C ≥220 mg/dl vs. non-HDL-C <100 mg/dl (*P* = 0.3687).

As demonstrated in Figure 2, Kaplan–Meier survival curves were diverged according to non-HDL-C stratification. The highest risk for all-cause mortality was observed when non-HDL-C <100 mg/dl, compared to other groups (all *p* < 0.005). Besides, more risk for cardiovascular mortality was only observed when non-HDL-C <100 mg/dl, compared to when non-HDL-C = 130-159 or 160-189 mg/dl (all *P* < 0.005). The optimal non-HDL-C concentration range was between 130 and 159 mg/dl for a lower risk of all-cause and cardiovascular death.

Table 2 summarised the multivariable Cox regression results. When non-HDL-C was treated as a continuous variable, per 30 mg/dl increment in non-HDL-C corresponded to the hazard ratio (HR) (95% confidence interval, CI) as 0.94 (95% CI 0.90–0.99, *p* = 0.0193) for all-cause mortality and 1.03 (95% CI 0.91–1.16, *p* = 0.6826) for cardiovascular mortality in model 3. When non-HDL-C was treated as a categorical variable, non-HDL-C = 130-159 mg/dl as a reference, the fully adjusted HRs for all-cause mortality were 1.98 (95% CI 1.59–2.48, *p* < 0.0001), 1.38 (95% CI 1.17–1.62, *p* = 0.0002), 1.11 (95% CI 0.95–1.31, *p* = 0.2007), 1.12 (95% CI 0.91–1.38, *p* = 0.2807) and 1.04 (95% CI 0.79–1.36, *p* = 0.8018) for non-HDL-C levels <100, 100–129, 160–189, 190–219, and ≥220 mg/dl, respectively. Meanwhile, for cardiovascular mortality, the fully adjusted HRs were 2.99 (95% CI 1.73–5.16, *p* < 0.0001), 1.72 (95% CI 1.10–2.69, *p* = 0.0169), 1.36 (95% CI 0.88–2.11, *p* = 0.1636), 1.61 (95% CI 0.94–2.73, *p* = 0.0811), and 2.16 (95% CI 1.17–3.98, *p* = 0.0134) for non-HDL-C levels <100, 100–129, 160–189, 190–219, and ≥ 220 mg/dl, respectively.

**Table 2:** Multivariable cox regression analysis of non-HDL-C with all-cause and cardiovascular mortality.

	**Model 1 HR (95% CI), *P***	**Model 2 HR (95% CI), *P***	**Model 3 HR (95% CI), *P***
**All-cause mortality**			
Non-HDL-C (per 30 mg/dl increment)	1.01 (0.97, 1.05) 0.6087	0.95 (0.91, 0.99) 0.0207	0.94 (0.90, 0.99) 0.0193
Non-HDL-C group, mg/dl			
<100	1.20 (0.97, 1.49) 0.0926	2.18 (1.76, 2.71) <0.0001	1.98 (1.59, 2.48) <0.0001
100–129	1.18 (1.00, 1.39) 0.0465	1.45 (1.22, 1.71) <0.0001	1.38 (1.17, 1.63) 0.0002
130–159	Reference	Reference	Reference
160–189	1.13 (0.96, 1.32) 0.1518	1.12 (0.95, 1.31) 0.1758	1.11 (0.95, 1.31) 0.2007
190–219	1.06 (0.87, 1.30) 0.5705	1.16 (0.95, 1.42) 0.1493	1.12 (0.91, 1.38) 0.2807
≥220	1.09 (0.85, 1.40) 0.4902	1.24 (0.97, 1.60) 0.0857	1.04 (0.79, 1.36) 0.8018
*P* for trend	0.3574	<0.0001	<0.0001
**Cardiovascular mortality**			
Non-HDL-C (per 30 mg/dl increment)	1.08 (0.98, 1.19) 0.1076	1.05 (0.94, 1.18) 0.3504	1.03 (0.91, 1.16) 0.6826
Non-HDL-C group, mg/dl			
<100	1.64 (0.97, 2.77) 0.0667	3.15 (1.85, 5.36) <0.0001	2.99 (1.73, 5.16) <0.0001
100–129	1.36 (0.88, 2.11) 0.1699	1.66 (1.07, 2.58) 0.0241	1.72 (1.10, 2.69) 0.0169
130–159	Reference	Reference	Reference
160–189	1.27 (0.83, 1.96) 0.2726	1.32 (0.86, 2.04) 0.2039	1.36 (0.88, 2.11) 0.1636
190–219	1.38 (0.83, 2.31) 0.2128	1.63 (0.97, 2.72) 0.0633	1.61 (0.94, 2.73) 0.0811
≥220	2.03 (1.18, 3.50) 0.0107	2.52 (1.45, 4.35) 0.0010	2.16 (1.17, 3.98) 0.0134
P for trend	0.5151	0.8498	0.5035

*Non-HDL-C, non-high-density lipoprotein cholesterol; HR, hazard ratio; CI, confidence interval*.

*Model 1: no adjustment; Model 2: adjusted for age and race; Model 3: adjusted for age, race, education, body mass index, systolic blood pressure, diastolic blood pressure, smoking, diabetes, hypertension, coronary heart disease, stroke, creatinine, haemoglobin, glycated haemoglobin A1c, triglycerides, energy intake, protein intake, carbohydrate intake, and total fat intake*.

### Non-Linear Relationships of Non-HDL-C With All-Cause and Cardiovascular Mortality

**Figure 3:**
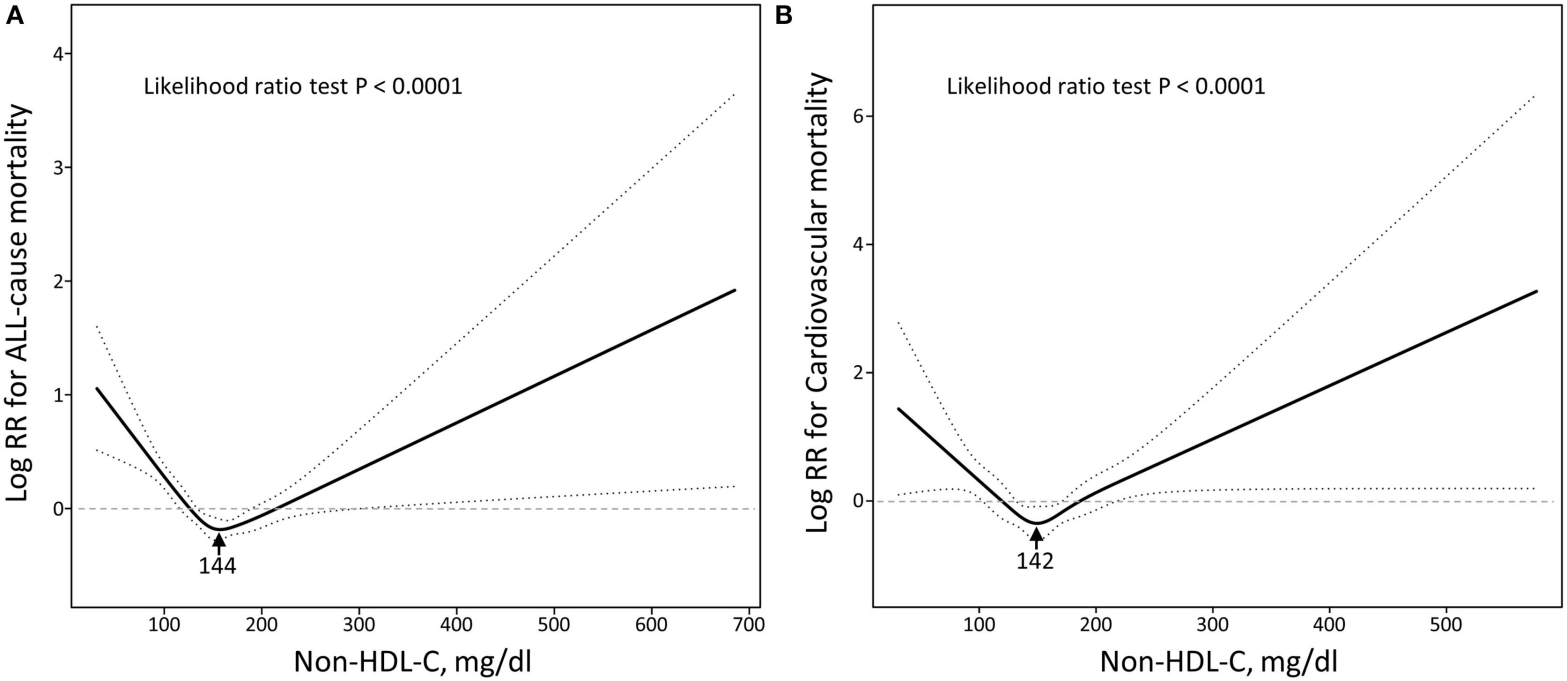
Restricted cubic spine models of non-HDL-C with all-cause **(A)** and cardiovascular **(B)** mortality. Non-HDL-C, non-high-density lipoprotein cholesterol. Restricted cubic spine models were adjusted for adjusted for age, race, education, body mass index, systolic blood pressure, diastolic blood pressure, smoking, diabetes, hypertension, coronary heart disease, stroke, creatinine, haemoglobin, glycated haemoglobin A1c, triglycerides, energy intake, protein intake, carbohydrate intake, and total fat intake.

As shown in Figure 3, the multivariable adjusted restrictive cubic curves confirmed that the relationships of non-HDL-C with all-cause and cardiovascular mortality were U-shaped (All *p* for likelihood ratio test <0.0001). The threshold values of non-HDL-C related to the lowest risk in multivariable adjusted analyses were 144 mg/dl for all-cause mortality and 142 mg/dl for cardiovascular mortality. As shown in Table 3, below the threshold, per 30 mg/dl increase in non-HDL-C reduced a 28% increased risk of all-cause mortality (*p* < 0.0001) and a 40% increased risk of cardiovascular mortality (*p* = 0.0037). Inversely, above the threshold, per 30 mg/dl increase in non-HDL-C accelerated risk of both all-cause mortality (HR 1.11, 95% CI 1.03–1.20, *p* = 0.0057) and cardiovascular mortality (HR 1.30, 95% CI 1.09–1.54, *p* = 0.0028).

**Table 3:** The results of two piecewise linear regression model of non-HDL-C with all-cause and cardiovascular mortality.

	**All-cause mortality** **HR (95% CI) *P***	**Cardiovascular mortality** **HR (95% CI) *P***
Cut-off value	144	142
<Cut-off value (as continuous variables, per 30 mg/dl increment)	0.72 (0.62, 0.82) <0.0001	0.60 (0.42, 0.85) 0.0037
≥Cut-off value (as continuous variables, per 30 mg/dl increment)	1.11 (1.03, 1.20) 0.0057	1.30 (1.09, 1.54) 0.0028

*Non-HDL-C, non-high-density lipoprotein cholesterol; HR, hazard ratio; CI, confidence interval*.

*The two piecewise linear regression models were adjusted for adjusted for age, race, education, body mass index, systolic blood pressure, diastolic blood pressure, smoking, diabetes, hypertension, coronary heart disease, stroke, creatinine, haemoglobin, glycated haemoglobin A1c, triglycerides, energy intake, protein intake, carbohydrate intake, and total fat intake*.

### Subgroups Analysis of the Risk of All-Cause and Cardiovascular Mortality

**Figure 4:**
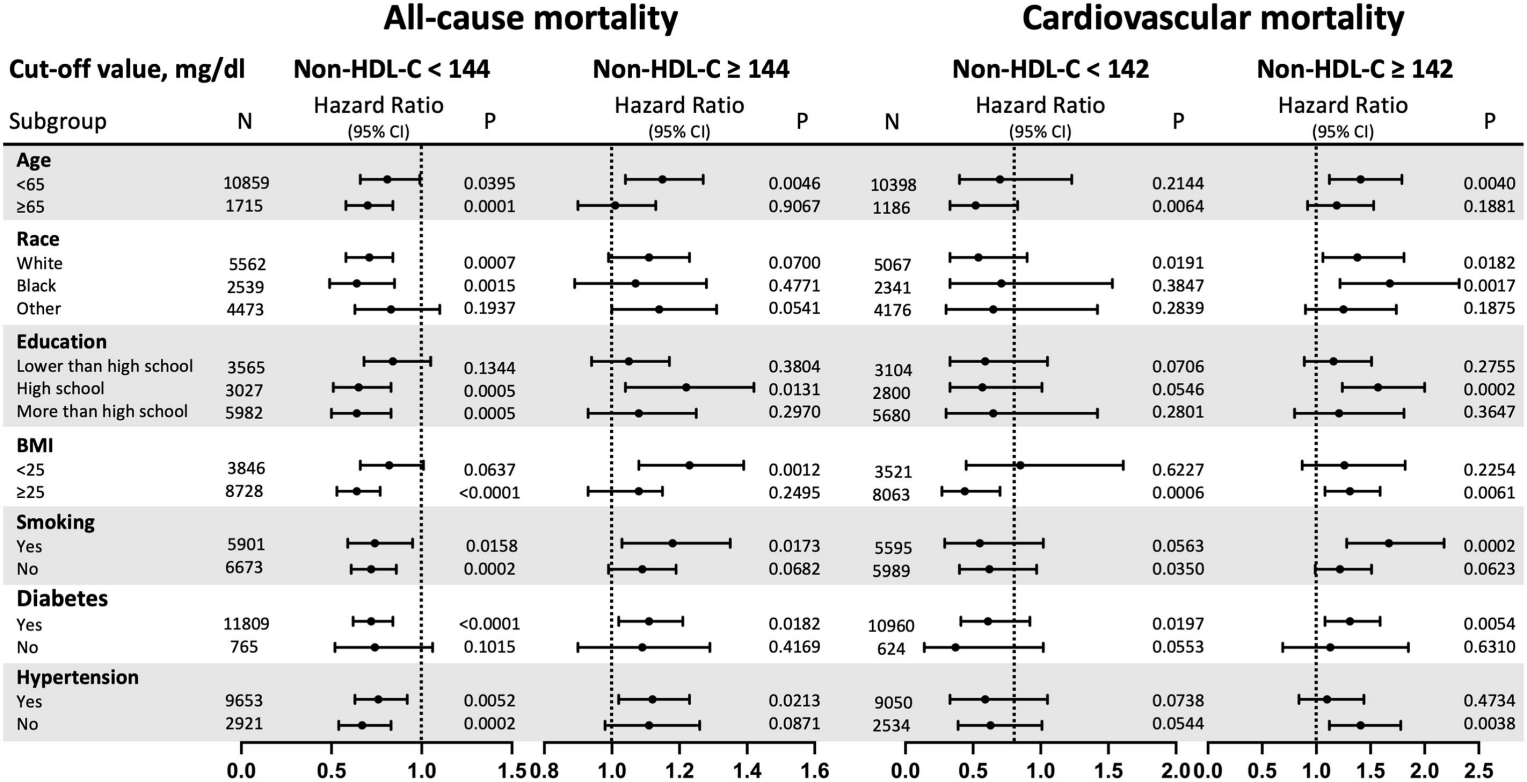
Subgroup analysis. Non-HDL-C, non-high-density lipoprotein cholesterol; HR, hazard ratio; CI, confidence interval; BMI, body mass index. Results are expressed as multivariable-adjusted HR in continuous analyses (Non-HDL-C per 30 mg/dl increment). When analysing a subgroup variable, age, race, education, BMI, systolic blood pressure, diastolic blood pressure, smoking, diabetes, hypertension, coronary heart disease, stroke, creatinine, haemoglobin, glycated haemoglobin A1c, triglycerides, energy intake, protein intake, carbohydrate intake, and total fat intake were all adjusted except the variable itself.

The stratified analyses are demonstrated in Figure 4 (Detail data as shown in Supplementary Table S1). The non-linear relationships for all-cause mortality with statistical significance were found among participants who were aged <65 years old, and race (White). Besides, the non-linear relationships for cardiovascular mortality with statistical significance were found among participants who were race (White), BMI ≥ 25 kg/m^2^, and without diabetes. When non-HDL-C ≥ 142 mg/dl, per 30 mg/dl increase in non-HDL-C increased risk of cardiovascular mortality were 1.41-fold for aged <65 years old (*p* = 0.0040), 1.38-fold for race (White) (*p* = 0.0182) and 1.68-fold for race (Black) (*p* = 0.0017), 1.57-fold for high school education (*p* = 0.0002), 1.67-fold for smoking (*p* = 0.0002), 1.31-fold for participants without diabetes (*p* = 0.0054) and 1.41-fold for participants with hypertension (*p* = 0.0038).

## Discussion

The novel finding of the study is that both low and high non-HDL-C levels were significantly associated with increased risk of all-cause and cardiovascular mortality among men without statin therapy in U-shaped relationships. Furthermore, we confirmed that the non-HDL-C level was related to the lowest risk of all-cause and cardiovascular mortality at threshold values of 144 and 142 mg/dl, respectively. These new results are probable to have implications for the interpretation of levels of non-HDL-C in clinical practise.

Global age-standardised mean non-HDL-C remained almost unchanged from 1980 to 2018, and high non-HDL-C was responsible for an estimated 3.9 million worldwide deaths from ischemic heart disease and ischemic stroke in 2017, accounting for a third of deaths from these causes (18). Undoubtedly, it is particularly urgent to clearly understand the relationship of non-HDL-C stratification with death, and locate the best threshold values of non-HDL-C. One of our major findings is that once non-HDL-C levels are greater than threshold values, is closely contributed to higher mortality. This finding is similar to previous several studies in different populations (8, 9, 11, 13, 19–21). The potential explanation for this finding is that extremely high non-HDL-C levels play a role in accelerated atherosclerosis, leading to an increased risk of death (22). In United States population study, non-HDL-C may be best suited for the prediction of future coronary artery calcium (CAC) progression, especially since non-HDL-C levels ≥ 190 mg/dl are consistently associated with significant CAC progression in the overall population (β 16.4, 95%CI −5.63 to 27.2, *p* = 0.003) (23). One genetic study finding is that levels of non-HDL-C are associated with the extent of coronary atherosclerosis. Besides, the mutations of some genes like LDLR, apolipoprotein B, and proprotein convertase subtilisin/kexin type 9 (PCSK9), can result in hypercholesterolemia, and guidelines suggested that non-HDL-C ≥ 220 mg/dl could possibly imply hereditary genetic hypercholesterolemia (11, 24). Patients with hypercholesterolemia have increased non-HDL-C and are more prone to suffer from atherosclerotic cardiovascular and cardiovascular death (11, 23).

Moreover, the present study contributed evidence that lower non-HDL-C is also closely related to higher mortality in men without statin therapy, and indicated a U-shaped association. Although the disparity in the study population, in accordance with our results, several previous studies have observed the U-shaped association between non-HDL-C and mortality (11, 12). One study by Cheng and colleagues analysing data from NHANES demonstrated that relatively higher or lower non-HDL-C concentrations were linked to increased mortality, and the lowest risk was found at threshold values of 158 and 190 mg/dl for all-cause and cardiovascular mortality, respectively. The difference in threshold estimates might be attributed to different study populations, of which all patients in the study by Cheng et al. were hypertension, and had relatively higher non-HDL-C levels (25). Likewise, the U-shaped relationships between non-HDL-C and the risk of all-cause and cardiovascular mortality have been shown in patients with CKD and the optimal non-HDL-C concentration range was between 116.2 and 143.9 mg/dl (11). Similarly, a study of a general population cohort also found a U-shaped association between levels of LDL-C and the risk of all-cause mortality (26). In patients with CHD, a paradoxical association existed between baseline non-HDL-C and long-term all-cause mortality, but disappeared after taking into account the effects of malnutrition, indicating that the worse long-term prognosis in the low non-HDL-C group (<2.2 mmol/L) was mainly mediated by the underlying effect of malnutrition (27). Apart from that, an inverse association between cholesterol and mortality has been demonstrated in the elderly (28, 29). Another population-based register study including 118,160 subjects without statin therapy found that high lipoprotein levels were associated with lower mortality indicating that high lipoprotein levels do not seem to be definitely harmful in the general population (29). Similarly, participants with low serum TC seem to have a lower survival rate than participants with an elevated cholesterol level, irrespective of concomitant diseases or health status (28). Unexpectedly, the finding of a U-shaped association in our study is inconsistent with a positive association in another study (13). The differences between the two studies in the population (United States or Israel), sample size (13,562 or 4,832), and follow-up time (98.38 ± 53.78 months or 22.1 ± 3.2 years) may result in different conclusions.

However, the underlying mechanism of U-shaped association is not clear. First, one possible reason is that the participants with the lowest cholesterol levels had a poorer health status (28), or debilitation and illness have been hypothesised to cause a decrease in levels of cholesterol (26, 30, 31). Second, higher HDL-C equals to low non-HDL-C levels according to the calculation formula, extremely high HDL-C increases mortality in the general population by analysing the data from NHANES (12). The genetic variation of particular genes and variation of the size or function of HDL particles may be the underlying mechanism (12). Finally, the U-shaped association between lipoprotein levels and mortality may be similar to the obesity paradox, which is largely explained by methodological issues, including reverse causation. No matter how, more studies are needed to clarify the exact mechanism of the U-shaped association.

The advantage of the present study lies in its relatively large sample size and long-term follow-up. Regarding clinical importance, our novel findings are conducive to understanding the risk stratification of non-HDL-C and remind us that when initiating lipid-lowering therapy in clinical practise, attention should be paid to assessing the absolute risk of atherosclerotic cardiovascular disease (26, 32, 33), rather than starting treatment based solely on a moderate increase in levels of a specific lipid marker. Anyway, there are still some limitations to this study. First, during long-term follow-up, only a single measurement of serum non-HDLC concentration at baseline is available, leading to potential bias and failure to evaluate the affection of non-HDL-C trajectories on mortality. Second, although we adjusted many relevant confounding variables that were considered to influence mortality, residual confounders and hidden comorbidities might have been not eliminated. Finally, our study was performed in a nationally representative sample of men in the United States, so our results may not be easily extrapolated to the population in other regions.

## Conclusion

From a population-based cohort study base on the national representative database, our study demonstrated that non-HDL-C was U-shaped and related to all-cause and cardiovascular mortality among men without statin therapy. The more clear risk stratification of non-HDL-C and comprehensive strategic management to deal with dyslipidemia deserves further investigation for confirmation.

## Data Availability Statement

The datasets presented in this study can be found in online repositories. The names of the repository/repositories and accession number(s) can be found below: https://www.cdc.gov/nchs/nhanes/index.htm.

## Ethics Statement

The studies involving human participants were reviewed and approved by the US Centres for Disease Control and Prevention ratified the study protocols. The patients/participants provided their written informed consent to participate in this study.

## Author Contributions

R-XZ, J-PX, Y-JK, L-HG, and M-ZZ: conceived and designed the study. R-XZ, J-PX, and J-WT: collected and analysed the data. R-XZ: drafted the paper. M-ZZ: revised the manuscript. All authors have reviewed the final manuscript. All authors contributed to the article and approved the submitted version.

## Funding

This work was partially supported by the National Natural Scientific Foundation (No. 82004135 to R-XZ), the Research Fund for Zhaoyang Talents of Guangdong Provincial Hospital of Chinese Medicine (No. ZY2022KY03 to R-XZ), and the Specific Research Fund for TCM Science and Technology of Guangzhou Provincial Hospital of Chinese Medicine (YN10101915 to M-ZZ).

## Conflict of Interest

The authors declare that the research was conducted in the absence of any commercial or financial relationships that could be construed as a potential conflict of interest.

## Publisher's Note

All claims expressed in this article are solely those of the authors and do not necessarily represent those of their affiliated organizations, or those of the publisher, the editors and the reviewers. Any product that may be evaluated in this article, or claim that may be made by its manufacturer, is not guaranteed or endorsed by the publisher.
