# Supplementary material for: U-Shaped Relationship of Non-HDL Cholesterol With All-Cause and Cardiovascular Mortality in Men Without Statin Therapy
Source: Front Cardiovasc Med. 2022 Jul 7;9:903481. doi: 10.3389/fcvm.2022.903481 (PMC9300868; doi:10.3389/fcvm.2022.903481)
Supplement: Supplementary file 1 [file Table_1.DOCX]

**TABLE S1** Data for Fiugre 4 (Subgroup analysis)

|  | N | All-cause mortality  HR (95% CI) P | | | N | Cardiovascular mortality  HR (95% CI) P | |
| --- | --- | --- | --- | --- | --- | --- | --- |
| Cut-off value, mg/dl |  | <144 | ≥144 | |  | <142 | ≥142 |
| **Age** |  |  |  | |  |  |  |
| <65 | 10859 | 0.81 (0.66, 0.99) 0.0395 | 1.15 (1.04, 1.27) 0.0046 | | 10398 | 0.70 (0.40, 1.23) 0.2144 | 1.41 (1.12, 1.79) 0.0040 |
| ≥65 | 1715 | 0.70 (0.58, 0.84) 0.0001 | 1.01 (0.90, 1.13) 0.9067 | | 1186 | 0.52 (0.33, 0.83) 0.0064 | 1.19 (0.92, 1.53) 0.1881 |
| **Race** |  |  |  | |  |  |  |
| White | 5562 | 0.71 (0.58, 0.86) 0.0007 | 1.10 (0.99, 1.23) 0.0700 | | 5067 | 0.54 (0.33, 0.90) 0.0191 | 1.38 (1.06, 1.81) 0.0182 |
| Black | 2539 | 0.64 (0.49, 0.85) 0.0015 | 1.07 (0.89, 1.28) 0.4771 | | 2341 | 0.71 (0.33, 1.53) 0.3847 | 1.68 (1.22, 2.32) 0.0017 |
| Other Race | 4473 | 0.83 (0.63, 1.10) 0.1937 | 1.14 (1.00, 1.31) 0.0514 | | 4176 | 0.69 (0.34, 1.37) 0.2839 | 1.25 (0.90, 1.74) 0.1875 |
| **Education** |  |  |  | |  |  |  |
| Lower than high school | 3565 | 0.84 (0.68, 1.05) 0.1344 | 1.05 (0.94, 1.17) 0.3804 | | 3104 | 0.59 (0.33, 1.05) 0.0706 | 1.16 (0.89, 1.51) 0.2755 |
| High school | 3027 | 0.65 (0.51, 0.83) 0.0005 | 1.22 (1.04, 1.42) 0.0131 | | 2800 | 0.57 (0.33, 1.01) 0.0546 | 1.57 (1.24, 2.00) 0.0002 |
| More than high school | 5982 | 0.64 (0.50, 0.82) 0.0005 | 1.08 (0.93, 1.25) 0.2970 | | 5680 | 0.65 (0.30, 1.42) 0.2801 | 1.21 (0.80, 1.81) 0.3647 |
| **Body mass index** |  |  |  | |  |  |  |
| <25 | 3846 | 0.82 (0.66, 1.01) 0.0637 | 1.23 (1.08, 1.39) 0.0012 | | 3521 | 0.85 (0.45, 1.61) 0.6227 | 1.26 (0.87, 1.82) 0.2254 |
| ≥25 | 8728 | 0.64 (0.53, 0.77) <0.0001 | 1.05 (0.96, 1.15) 0.2495 | | 8063 | 0.44 (0.27, 0.70) 0.0006 | 1.31 (1.08, 1.59) 0.0061 |
| **Smoking** |  |  |  | |  |  |  |
| NO | 5901 | 0.74 (0.59, 0.95) 0.0158 | 1.18 (1.03, 1.35) 0.0173 | | 5595 | 0.55 (0.29, 1.02) 0.0563 | 1.67 (1.28, 2.18) 0.0002 |
| YES | 6673 | 0.72 (0.61, 0.86) 0.0002 | 1.09 (0.99, 1.19) 0.0682 | | 5989 | 0.62 (0.40, 0.97) 0.0350 | 1.22 (0.99, 1.51) 0.0623 |
| **Diabetes** |  |  |  | |  |  |  |
| NO | 11809 | 0.72 (0.62, 0.84) <0.0001 | 1.11 (1.02, 1.21) 0.0182 | | 10960 | 0.61 (0.41, 0.92) 0.0197 | 1.31 (1.08, 1.59) 0.0054 |
| YES | 765 | 0.74 (0.52, 1.06) 0.1015 | 1.08 (0.90, 1.29) 0.4169 | | 624 | 0.37 (0.14, 1.02) 0.0553 | 1.13 (0.69, 1.85) 0.6310 |
| **Hypertension** |  |  |  | |  |  |  |
| NO | 9653 | 0.76 (0.63, 0.92) 0.0052 | 1.12 (1.02, 1.23) 0.0213 | | 9050 | 0.59 (0.33, 1.05) 0.0738 | 1.10 (0.84, 1.44) 0.4734 |
| YES | 2921 | 0.67 (0.54, 0.83) 0.0002 | | 1.11 (0.98, 1.26) 0.0871 | 2534 | 0.63 (0.39, 1.01) 0.0544 | 1.41 (1.12, 1.78) 0.0038 |

Non-HDL-C, non-high-density lipoprotein cholesterol; HR, hazard ratio; CI, confidence interval; BMI, body mass index; HbA1c, glycated hemoglobin A1c.

Results are expressed as multivariable-adjusted HR in continuous analyses (Non-HDL-C per 30 mg/dl increment). When analyzing a subgroup variable, age, race, education, body mass index, systolic blood pressure, diastolic blood pressure, smoking, diabetes, hypertension, coronary heart disease, stroke, creatinine, hemoglobin, HbA1c, triglycerides, energy intake, protein intake, carbohydrate intake, and total fat intake were all adjusted except the variable itself.
